# Interaction of VvDELLA2 and VvCEB1 Mediates Expression of Expansion-Related Gene during GA-Induced Enlargement of Grape Fruit

**DOI:** 10.3390/ijms241914870

**Published:** 2023-10-03

**Authors:** Zhenhua Liu, Yan Wang, Pingyin Guan, Jianfang Hu, Lei Sun

**Affiliations:** 1 Institute of Forestry and Pomology, Beijing Academy of Agriculture and Forestry Sciences, Beijing Engineering Research Center for Deciduous Fruit Trees, Beijing 100093, China; liuzhenhua93@163.com (Z.L.); wangyanifp@baafs.net.cn (Y.W.); 2 Beijing Engineering Research Center for Deciduous Fruit Trees, Beijing 100093, China; 3 College of Horticulture, China Agricultural University, Beijing 100193, China; pyguan@cau.edu.cn

**Keywords:** GA_3_, cell expansion, VvDELLA2, VvCEB1, grape, early fruit enlargement

## Abstract

Exogenous gibberellin treatment can promote early growth of grape fruit, but the underlying regulatory mechanisms are not well understood. Here, we show that VvDELLA2 directly regulates the activity of the VvCEB1 transcription factor, a key regulator in the control of cell expansion in grape fruit. Our results show that VvCEB1 binds directly to the promoters of cell expansion-related genes in grape fruit and acts as a transcriptional activator, while VvDELLA2 blocks VvCEB1 function by binding to its activating structural domain. The exogenous gibberellin treatment relieved this inhibition by promoting the degradation of VvDELLA2 protein, thus, allowing VvCEB1 to transcriptionally activate the expression of cell expansion-related genes. In conclusion, we conclude that exogenous GA_3_ treatment regulates early fruit expansion by affecting the VvDELLA-VvCEB1 interaction in grape fruit development.

## 1. Introduction

Early fruit development is very important for fruit production. Phytohormones play an important role in early fruit development [1]. Phytohormones, such as auxin and gibberellin (GA), are the main regulatory factors that control the fruit enlargement of different plant species. Auxin and GA work together to promote cell division and expansion, thus, regulating fruit development and enlargement after fertilization. The function of auxin in fruit growth is GA-dependent in Arabidopsis, tomato, and grape [2]. One of the main hormones that promote early fruit cell expansion is gibberellin (GA) [1]. In tomato fruit cell expansion, the expression of *GA3ox* and *GA20ox* in fruit tissue is upregulated and the bioactive GA concentration increases [3,4]. Expression of the *PslGAI*, *PslRGL*, and *PslRGA* genes, which encode three DELLA-like proteins, was low during plum fruit cell expansion, while *PslRGL* and *PslRGA* expression peaked at the peak of fruit cell division, when GA levels remained relatively low [5]. Pre-flowering GA_3_ application to grapes increased the fruit set, induced parthenocarpy, and promoted early fruit development [6,7], and exogenous GA_3_ treatment also increased endogenous gibberellin content and caused significant differences in the expression of genes related to GA biosynthesis and its signal transduction pathway compared to the control [8]; this suggests that fruit cell expansion requires the involvement of gibberellin or GA signaling [9].

The promotion of fruit cell expansion by GAs is generally thought to be related to the fact that GA stimulates cell wall relaxation in plants [10]. GAs usually relax the cell wall by inducing the expression of expansion-related proteins and xyloglucan endoglucanases/endohydrolases (*XTHs*) genes, which leads to changes in the cell wall that allow cell expansion [11,12,13,14]. Expansins (EXPs) are cell wall relaxases that are involved in regulating cell wall expansion and enlargement in a pH-dependent manner [15]. In plants, there are four distinct EXP families, namely expansin A (EXPA), expansin B (EXPB), expansin A-like (EXLA), and expansin B-like (EXLB). EXPAs and EXPBs are known to be involved in the expansion and modification of plant cell walls, while the specific functions of EXLAs and EXLBs are currently unknown. Grapes experienced a significant upregulation in cell wall relaxation-related genes (*EXPAs*, *XETs*) in fruit after GA_3_ treatment, suggesting that cell wall relaxation genes (*EXPAs*, *XETs*) play a key role in GA_3_-induced grape fruit expansion [8,16].

In recent years, the molecular mechanisms of the gibberellin signaling cascade have been well described. The gibberellin response pathway is negatively regulated by its central repressor, the DELLA protein, a transcriptional regulator localized in the nucleus [17,18]. GA and its nuclear receptor, GIBBERELLIN INSENSITIVE DWARF1 (GID1), binding enhances the GID1-DELLA interaction, leading to rapid degradation of DELLAs proteins via the ubiquitin–proteasome pathway, thereby relieving the growth inhibition caused by DELLA [19]. There are five *DELLA* genes in Arabidopsis, all mutants of which lead to parthenocarpy and promote early siliqua elongation, with effects similar to the length of siliqua obtained after GA_3_ treatment [20,21]. This result indicates that GA_3_ treatment or DELLA protein deficiency promotes early siliqua growth. Similarly, in tomato, mutations and RNAi downregulation of the sole *SlDELLA* gene (*PROCERA*) lead to parthenocarpy and promote early fruit growth [22,23,24,25]. There are three *VvDELLA1* (*VvGAI1*), *VvDELLA2*, and *VvDELLA3* genes in grapes, of which *VvGAI1* mainly regulates internode elongation and fruit set, but has no effect on berry size [26], while *VvDELLA2* is specifically expressed in fruit development [27], suggesting that *VvDELLA2* plays an important role in grape fruit development.

Feng et al. [28] found that DELLAs do not show any direct DNA binding by chromatin immunoprecipitation (ChIP). This suggests that the function of the DELLA protein depends on protein-protein interactions that have direct or indirect effects on transcription [29,30]. It is thought that DELLA protein can interact with transcription factors (TFs) or chromatin remodeling factors (CRFs) to block the transcriptional activation of downstream target genes by these TFs [31,32]. In addition, DELLA proteins interact with other proteins by acting as transcriptional coactivators, activating growth-repressing transcription [33,34]. Alternatively, DELLA proteins can also form complexes with TRs to repressing growth-promoting transcription [35,36].

In plants, basic helix-loop-helix (bHLH) proteins act as transcription factors to regulate many growth and developmental processes. For example, they regulate fruit dehiscence, carpel and epidermal development, stress responses, photosensitive pigment signaling, and secondary metabolic pathways, and are involved in the determination of plant organ size [37,38,39,40,41,42,43]. In grapes, *Vitis vinifera* cell elongation bHLH protein (VvCEB1) is a basic helix-loop-helix (bHLH) transcription factor that is specifically expressed during fruit enlargement and regulates berry cell expansion [43]. Overexpression of VvCEB1 in grapevine somatic embryos, Arabidopsis, and *N. benthamiana* resulted in enlarged plant cells [42,43]. This suggests that the VvCEB1 transcription factor plays an important role in grapevine fruit cell enlargement. However, it is not clear whether there is a link between the VvCEB1 transcription factor and the DELLA protein.

In this study, we reported that exogenous GA_3_ mediates the inhibitory effect of VvDELLA2 on VvCEB1 to regulate early grape fruit enlargement. Yeast two-hybrid (Y2H) and bimolecular fluorescence complementation (BIFC) show that VvDELLA2 interacts with VvCEB1 by binding to its activation domain. Electrophoretic mobility shift assay (EMSA) and luciferase reporter assay showed that VvCEB1 interacted with the promoters of *VvEXPA8* and *VvEXPA11* and activated the expression of the *LUC* reporter gene; however, this interaction is prevented by VvDELLA2 as the LUC activity was inhibited in the presence of VvDELLA2. Our data show that the interaction between VvDELLA2 and VvCEB1 inhibits the activation of downstream target genes by VvCEB1. In addition, the application of exogenous GA_3_ promotes the degradation of VvDELLA2 protein, indicating that exogenous GA_3_ induces the degradation of VvDELLA2 in early fruits to relieve the inhibitory effect of VvCEB1, promotes the activation of VvCEB1 on downstream cell expansion related genes *VvEXPA8* and *VvEXPA11*, and causes early enlargement of grape fruit.

## 2. Results

### 2.1. Effect of GA_3_ Treatment on the Growth of Berries at Early Fruit Development

**Figure 1:**
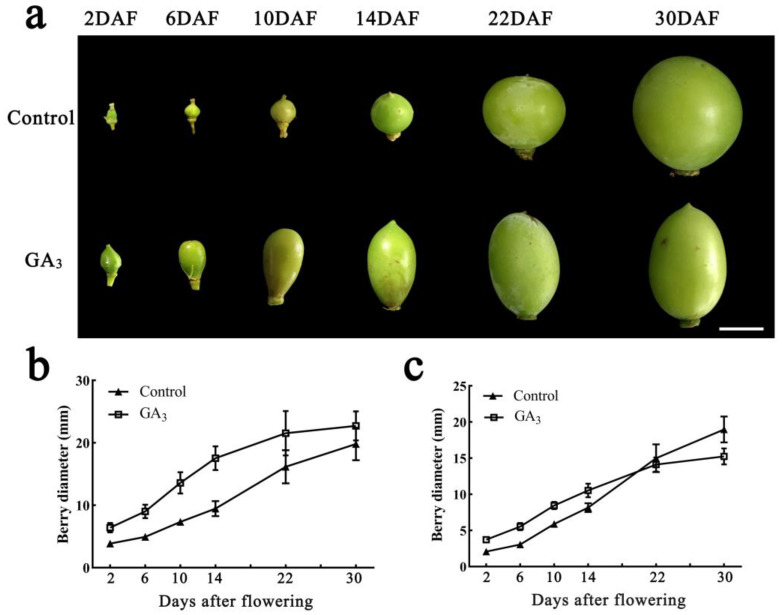
Effect of GA_3_ treatment on the shape and size in early fruit development of ‘Fenghou’. (**a**) Photos are representative of untreated control and GA_3_—treated fruits at 2, 6, 10, 14, 22, and 30 DAF; (**b**) changes in the longitudinal diameter of early fruit development; (**c**) changes in the transverse diameter of early fruit development. DAF represents days after flowering. The vertical bars represent the mean of three replicates ± SE. Data are mean ± SE (*n* = 3). Scale bars = 1 cm.

To determine the effect of GA_3_ treatment on early fruit development, measurements (transverse diameter and longitudinal diameter) were carried out 2, 6, 10, 14, 22, and 30 d after flowering (DAF) in the ‘Fenghou’ grapes. As shown in Figure 1a, the fruit enlarged rapidly after GA_3_ treatment and the longitudinal diameter from GA_3_-treated fruit was significantly higher than that of the control. The longitudinal diameter and transverse diameter from GA_3_-treated fruit were significantly higher than those of the control fruit from 2 to 14 DAF (Figure 1b,c). From 22 to 30 DAF, although the longitudinal diameter of the GA_3_-treated fruit was still larger than that of the control fruit of the same period, the difference with the control became smaller (Figure 1b), while the transverse diameter of the GA_3_-treated fruit was significantly lower than in the control of the same period (Figure 1c).

### 2.2. Effect of Exogenous Gibberellins on the Anatomical Structure of the Fruit

**Figure 2:**
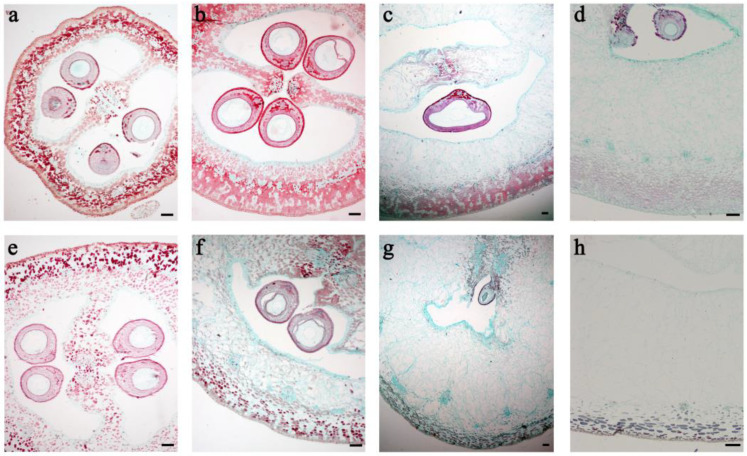
Effect of exogenous gibberellins on the anatomical structure of ‘Fenghou’ grapes. (**a**–**d**) Pericarp cells from untreated control fruits a t2, 6, 10, and 14 DAF; (**e**–**h**) pericarp cells from GA_3_-treated fruits at 2, 6, 10, and 14 DAF. DAF represents days after flowering. Scale bars = 100 μm.

To determine the effects of GA_3_ treatment on fruit structure and pericarp cells in grape, paraffin sections of GA_3_-treated and untreated ‘Fenghou’ young fruits from 2 to 14 DAF were stained with safranin and fast green and observed using an optical microscope. The ovary wall and mesocarp was slightly greater in the GA_3_-treated ovary than in the control at 2 DAF (Figure 2a,e). At 6 DAF, the mesocarp of the GA_3_-treated ovary began to turn into parenchyma cells and the ovary wall became significantly thicker (Figure 2b,f). At 10 and 14 DAF, the exocarp cells in the control fruit were tightly arranged and some of the mesocarp cells became parenchyma cells (Figure 2c,d). After GA_3_ treatment, all mesocarp cells became parenchyma cells (Figure 2g,h) and, especially at 14 DAF, only a few layers of exocarp cells near the epidermis were tightly arranged, while the rest developed into large, irregular parenchyma cells (Figure 2h).

**Figure 3:**
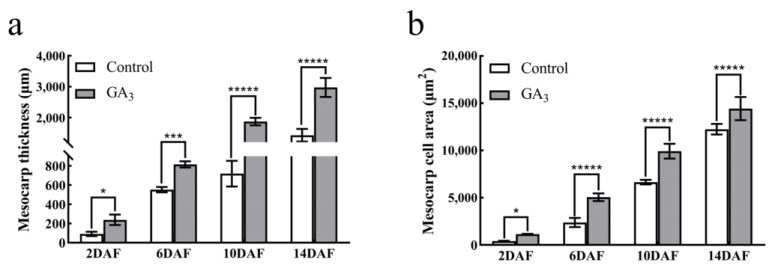
Effect of exogenous gibberellin on the pericarp tissue of ‘Fenghou’ grapes. (**a**) Mesocarp thickness; (**b**) mesocarp cell size. DAF represents days after flowering. Significance analysis was conducted with two-tailed Student’s *t*-tests (* *p* < 0.05; *** *p* < 0.001; ***** *p* < 0.00001). The vertical bars represent the mean of three replicates ± SE.

Furthermore, we carried out statistical analyses of the thickness of the exocarp, mesocarp, and endocarp, the number of cell layers, and the cell size of the young fruit from 2 to 14 DAF. The thickness of the exocarp and endocarp of GA_3_-treated and control fruits did not differ significantly at different developmental periods, but the mesocarp thickness of GA_3_-treated fruits was significantly greater than that of the control (Figure S1a,b and Figure 3a). Moreover, there was no significant difference in the number of cell layers in the exocarp, mesocarp, and endocarp between GA_3_-treated and control fruits (Figure S1c–e). Meanwhile, the cell sizes of the exocarp and endocarp did not differ significantly (Figure S1f–g), but the exogenous GA_3_-treated fruit had a significantly larger mesocarp cell area from 2 DAF than the control fruit developed at the same time (Figure 3b).

### 2.3. Effect of Exogenous GA_3_ on the Gene Expression Level of VvEXPAs

**Figure 4:**
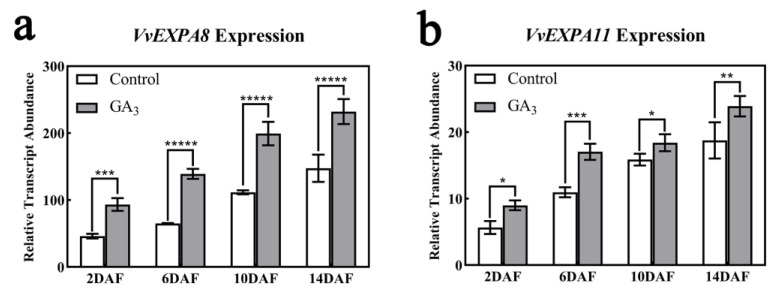
Effect of exogenous GA_3_ treatment on the expression levels of *VvEXPA8* and *VvEXPA11* genes. (**a**,**b**) Changes in expression of *VvEXPA8* and *VvEXPA11* after GA_3_ treatment, respectively. The expression levels of *VvEXPA8* and *VvEXPA11* were normalized against that of *VvUBQ*. DAF represents days after flowering. Significance analysis was conducted with two-tailed Student’s t-tests (* *p* < 0.05; ** *p* < 0.01; *** *p* < 0.001; ***** *p* < 0.00001). There were 3 replicates within each trial, and each experiment was repeated at least 3 times. The vertical bars represent the mean of three replicates ± SE.

As a result of exogenous GA_3_ treatment, mesocarp cells were significantly enlarged. In this regard, we determined the expression levels of the cell expansion-related genes *VvEXPA8* and *VvEXPA11* (involved in cell wall expansion) after GA_3_ treatment and found that *VvEXPA8* and *VvEXPA11* were significantly higher than the control (Figure 4a,b). This suggests that exogenous GA_3_ treatment may relax the cell wall of pericarp cells by promoting the expression of the cell wall relaxation genes *VvEXPA8* and *VvEXPA11*, which in turn leads to cell expansion.

### 2.4. Effect of Exogenous GA_3_ on the Gene Expression of VvDELLA2 and Its Protein Level

**Figure 5:**
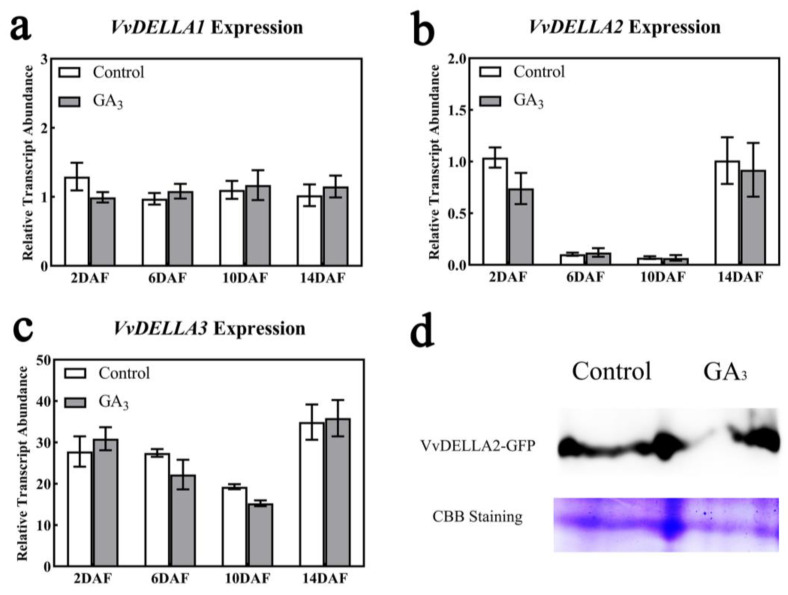
Effect of exogenous GA_3_ treatment on the expression pattern of *VvDELLAs* genes and VvDELLA2 protein. (**a**) Changes in the expression of *VvDELLA1* after gibberellin treatment; (**b**) changes in the expression of *VvDELLA2* after gibberellin treatment; (**c**) changes in the expression of *VvDELLA3* after gibberellin treatment; (**d**) changes in the expression of VvDELLA2 protein after gibberellin treatment. The expression levels of *VvDELLA1, VvDELLA2*, and *VvDELLA3* were normalized against that of *VvUBQ*. DAF represents days after flowering. There were 3 replicates within each trial, and each experiment was repeated at least 3 times. The vertical bars represent the mean of three replicates ± SE.

As the DELLA protein is a key node in the GA pathway, we determined the changes in the expression levels of *VvDELLAs* genes and proteins at the young fruit stage after exogenous GA_3_ treatment and found that the *VvDELLA1* gene expression was slightly lower than the control at 2 days after treatment, and the rest of the *VvDELLA1* gene expression was higher than the control at 6, 10, and 14 days after treatment (Figure 5a). The *VvDELLA2* gene expression was lower than the control at both 2 and 14 days after GA_3_ treatment, and, in particular, the expression of the *VvDELLA2* gene was significantly lower at 6 and 10 days after treatment, both for the treatment and the control (Figure 5b). The *VvDELLA3* expression was slightly higher than the control at 2 and 14 days after GA_3_ treatment, while *VvDELLA3* expression was lower than the control at 6 and 10 days after GA_3_ treatment (Figure 5c). The gene expression indicated that *VvDELLA2* may play a major role in early fruit development. In this regard, the grapevine cotyledons were used to analyze whether VvDELLA2 protein was affected by exogenous GA_3_, and the results showed that VvDELLA2 protein expression was significantly lower after exogenous gibberellin treatment than in the control (Figure 5d and Figure S2).

### 2.5. GA Signaling Repressor VvDELLA2 Interacts with the Transcription Factor VvCEB1

**Figure 6:**
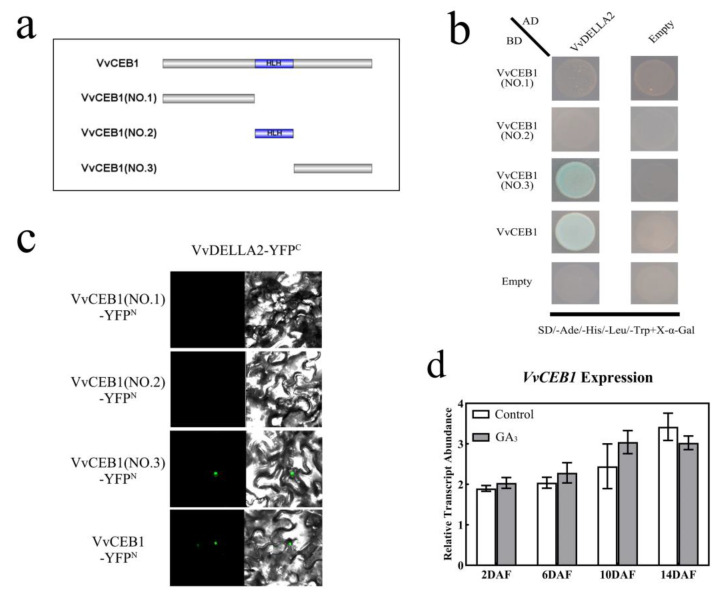
Physical interaction of VvDELLA2 with VvCEB1 in vitro and in vivo. (**a**) Schematic diagram showing the domain structures of VvCEB1 and its various truncated segments. (**b**) Yeast two-hybrid assays showing the interaction of VvDELLA2 with VvCEB1 and its truncated segments. Transformed yeast cells were grown on SD/-Trp/-Leu/-His/-Ade medium. AD, activation domain; BD, DNA-binding domain. (**c**) BiFC assay showing the interaction between VvDELLA2-YFP^C^ with VvCEB1-YFP^N^ and its truncated segments-YFP^N^ in *N. benthamiana* leaf nuclei. (**d**) Relative expression of *VvCEB1* after exogenous GA_3_ treatment. The expression levels of *VvCEB1* were normalized against that of *VvUBQ*. DAF represents days after flowering. There were 3 replicates within each trial, and each experiment was repeated at least 3 times. The vertical bars represent the mean of three replicates ± SE.

DELLA proteins do not possess any known DNA-binding domain and mainly interact with transcription factors to regulate gibberellin-mediated plant development [27]. Previous studies have shown that the transcription factor VvCEB1 in the grapes is involved in fruit cell expansion [43]. Meanwhile, analysis of *VvCEB1* gene expression at the young fruit enlargement stage showed that the GA_3_ treatment resulted in higher expression than the control (Figure 6d). Hence, we hypothesized that VvDELLA2 interacts with VvCEB1 to jointly regulate early grape fruit enlargement. To test whether VvDELLA2 and VvCEB1 directly interact, we performed a Y2H assay. We found that VvDELLA2 indeed interacted with VvCEB1 in this assay (Figure 6b). Moreover, the C terminal of VvCEB1 was sufficient and necessary for this interaction (Figure 6a,b). Bimolecular fluorescence complementation (BiFC) assay showed that VvDELLA2-YFP^C^ and VvCEB1-YFP^N^ interacted in the nuclei of living *N. benthamiana* cells (Figure 6c). These results support the hypothesis that gibberellin might regulate early grape fruit enlargement via the interaction of VvDELLA2 and VvCEB1.

### 2.6. VvCEB1 Activates VvEXPA8 and VvEXPA11 Gene Expression

**Figure 7:**
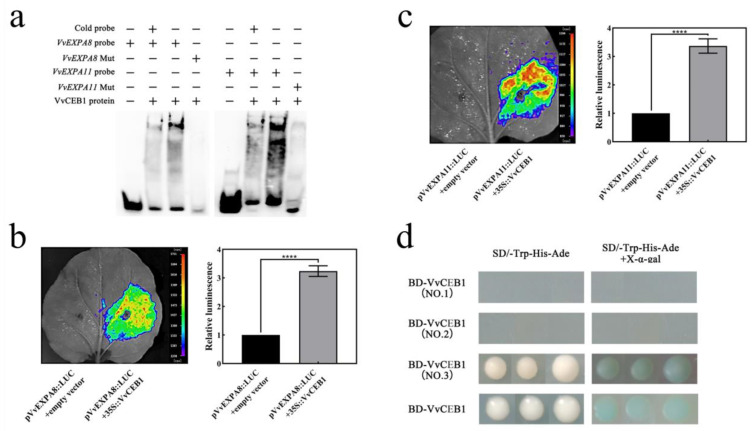
VvCEB1 activates the expression of *VvEXPA8* and *VvEXPA11*. (**a**) EMSA of VvCEB1 purified proteins using *VvEXPA8* and *VvEXPA11* probes; (**b**,**c**) luciferase assay of the *35S::VvCEB1* overexpression vector combined to *pVvEXPA8::LUC* and *pEXPA11::LUC*; (**d**) transcriptional activation assay of VvCEB1 full-length and segmentation in yeast. Significance analysis was conducted with two-tailed Student’s t-tests (**** *p* < 0.0001). There were 3 replicates within each trial, and each experiment was repeated at least 3 times. The vertical bars represent the mean of three replicates ± SE.

The above study showed that exogenous GA_3_ treatment promoted *VvEXPA8* and *VvEXPA11* gene expression (Figure 4), so we analyzed the relationship between the transcription factor VvCEB1 and the *VvEXPA8* and *VvEXPA11* genes. Our analysis of the *VvEXPA8* and *VvEXPA11* gene promoters revealed the presence of VvCEB1 binding sites at the *VvEXPA8* and *VvEXPA11* promoters, and the results of the EMSA assay showed that the VvCEB1 protein was able to form a complex with the DNA probe of the G-box on the *VvEXPA8* and *VvEXPA11* promoters. When non-biotin-labelled competing probes are present, the binding band was significantly weakened; when the probe was mutated, the binding band disappeared completely (Figure 7a), indicating that VvCEB1 can bind to the *VvEXPA8* and *VvEXPA11* promoters in vitro. The results of the luciferase assay showed that VvCEB1 could activate the expression of the reporter gene *LUC* linked to the *VvEXPA8* and *VvEXPA11* promoters, respectively (Figure 7b,c), indicating that the *VvEXPA8* and *VvEXPA11* promoters were regulated by VvCEB1. It was shown that VvCEB1 can activate the expression of *VvEXPA8* and *VvEXPA11* genes.

### 2.7. VvDELLA2 Inhibit the Activation of Downstream Target Genes by VvCEB1

**Figure 8:**
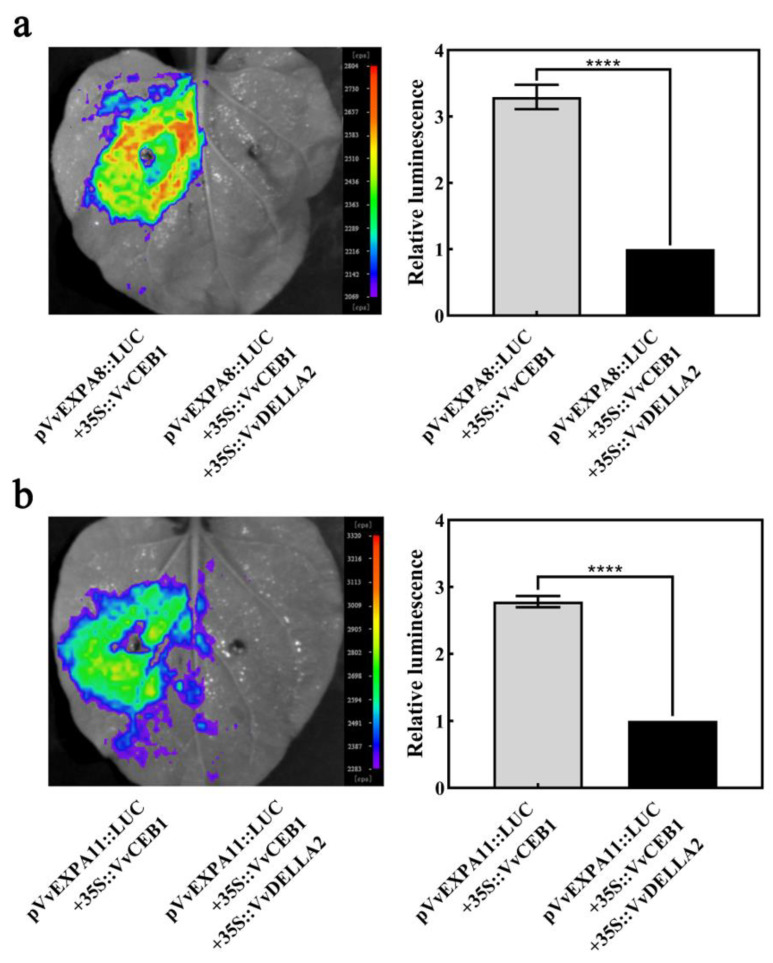
*VvEXPA8* and *VvEXPA11* promoters are regulated by the VvDELLA2-VvCEB1 complex. (**a**,**b**) Luciferase assay of co-expression of *35S::VvCEB1* and *35S::VvDELLA2* overexpression vectors combined to *pVvEXPA8::LUC* and *pVvEXPA11::LUC*, respectively. Significance analysis was conducted with two-tailed Student’s *t*-tests (**** *p* < 0.0001). There were 3 replicates within each trial, and each experiment was repeated at least 3 times. Values are the means ± SE.

Since VvCEB1 can activate *VvEXPA8* and *VvEXPA11* gene expression and there is an interaction between VvDELLA2 and VvCEB1 full-length and C-terminus. VvCEB1 and its C-terminus show transcriptional activation activity in yeast (Figure 7d). These results lead us to suspect that the interaction between VvDELLA2 and VvCEB1 affects the regulation of VvCEB1 on *VvEXPA8* and *VvEXPA11* genes. The results of the luciferase assay show that when only VvCEB1 is expressed, the expression of the reporter gene *LUC* linked to the *VvEXPA8* and *VvEXPA11* promoters can be activated (Figure 8a,b). When VvDELLA2 is co-expressed with VvCEB1, the expression of the reporter gene *LUC* linked to the *VvEXPA8* and *VvEXPA11* promoter is inhibited (Figure 8a,b), which indicates that VvDELLA2 inhibited the transcriptional activation of VvCEB1 by binding to the C-terminus of VvCEB1.

## 3. Discussion

**Figure 9:**
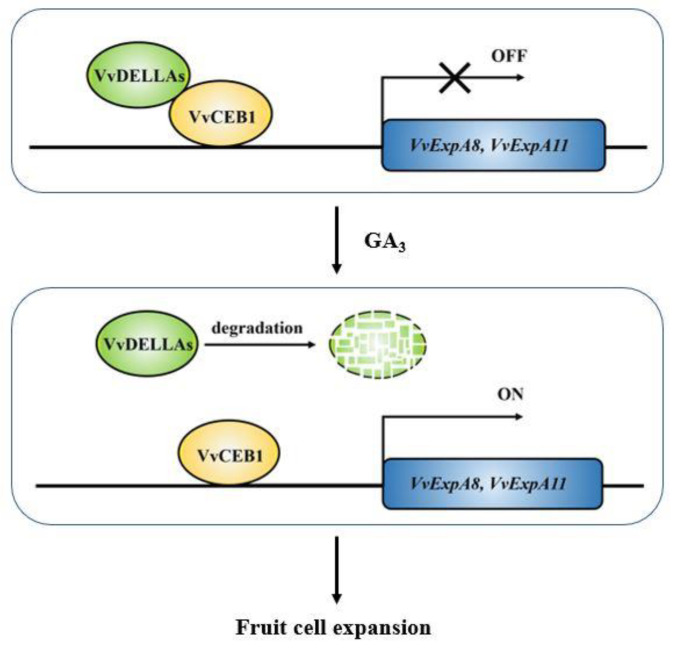
Pattern of exogenous GA_3_ regulation of cell expansion in early fruit development. *VvEXPA8* and *VvEXPA11* are functional genes that regulate cell wall relaxation, and their elevated expression promotes pericarp cell expansion. Prior to GA_3_ treatment, VvDELLA2 formed a complex with VvCEB1 that suppressed the expression of *VvEXPA8* and *VvEXPA11*. Upon exogenous GA_3_ treatment, VvDELLA2 protein was degraded and the repression of VvCEB1 transcriptional activity by VvDELLA2 was relieved, leading to the activation of *VvEXPA8* and *VvEXPA11* expression by VvCEB1 and contributing to cell expansion.

Based on our experimental findings, it can be inferred that the interplay between VvDELLA2 and VvCEB1 is responsible for facilitating the effects of exogenous gibberellin in stimulating the initial growth of grape berries during their early stages (see Figure 9). VvCEB1 activates the expression of genes (*VvEXPA8* and *VvEXPA11*) encoding expansion proteins (Figure 7). VvEXPA8 and VvEXPA11 are known to regulate cell wall relaxation and to promote cell expansion. This is because VvDELLA2 and VvCEB1 interact directly (Figure 6), and the interaction between VvDELLA2 and VvCEB1 inhibits the activation of their downstream cell expansion-related genes (Figure 8). However, after exogenous GA_3_ treatment, the VvDELLA2 protein was degraded (Figure 5d), which relieved the inhibition of VvCEB1 transcriptional activity, allowing VvCEB1 to activate the expression of *VvEXPA8* and *VvEXPA11*. In addition, we also found that VvDELLA2 interacts with the activation domain of VvCEB1, but not its DNA binding domain (Figure 6b,c and Figure 7d), which indicates that the VvDELLA2-VvCEB1 interaction may affect the transcriptional activation activity of VvCEB1. According to existing reports, most DELLA proteins interact with the DNA-binding domain of transcription factors to form an inactive complex, which prevents the transcription factors from binding to downstream target gene promoters and, thus, fails to activate the expression of target genes [28,30,31]. In summary, our results show that the role of the VvDELLA2-VvCEB1-VvEXPAs module in regulating exogenous GA_3_ to promote cell expansion in early fruit development is achieved through the following steps: (i) VvCEB1 binds to the *VvEXPAs* promoter and activates its expression (Figure 7a,c); (ii) VvDELLA2 interacts with the activation domain of VvCEB1, inhibiting VvCEB1’s transcriptional activation of *VvEXPAs* (Figure 6b,c, Figure 7d and Figure 8); (iii) treatment with exogenous GA_3_ promotes the degradation of VvDELLA2 protein, causes the inhibitory effect of VvDELLA2 on VvCEB1, and promotes the expression of *VvEXPAs* (Figure 5d and Figure 9).

During the fruit development stage, many factors affect changes in fruit quality. For example, in the early stages of fruit development, photosynthesis provides sugar/starch and other metabolites needed for fruit growth [44]. Phytohormones, including auxin, CK, and GA, play important roles in the early growth stages of fruit [45,46,47]. These phytohormones affect fruit size primarily by regulating cell division and expansion. Studies have shown that exogenous GA_3_ treatment can obviously promote early enlargement of grape berries. Cheng et al. [6] used exogenous GA_3_ to treat three grape varieties, namely ‘Kyoho’, ‘Red Globe’, and ‘Thompson Seedless’, and found that during the early fruit development period, the transverse and longitudinal diameters of the GA_3_-treated fruit were significantly higher than those of the untreated grapes; interestingly, in the seeded varieties ‘Kyoho’ and ‘Red Globe’, the transverse diameter of the GA_3_-treated fruit became significantly smaller than the untreated fruit after 15 DAF, while in the seedless variety ‘Thompson Seedless’, the transverse and longitudinal diameters of GA_3_-treated fruits were always larger than those of untreated fruits. In this study, we treated ‘Fenghou’ with exogenous GA_3_, and we also observed similar results to the above-mentioned seeded grapes: in the range of 2 to 14 DAF, the transverse and longitudinal diameters of the GA_3_-treated fruits were larger than those of the control, while at 22 DAF and later, the transverse diameter of the treated fruit was smaller than that of the control fruit. This shows that for seeded grape varieties, GA_3_ treatment plays an important role in early fruit development.

The fruit size mainly depends on the cell division and expansion. The regulation of cell division has been shown to be affected by auxin signals [9], while the regulation of cell expansion is not well understood at present. In this study, we found that the area of mesocarp cells in young fruits after GA_3_ treatment was significantly larger than that in the control, but there was no significant difference in the number of cell layers. This shows that the exogenous GA_3_ treatment mainly affects the expansion of cells rather than the number of cells. This is consistent with the results of previous studies [48]. Studies on the related transcriptome changes after GA_3_ treatment have shown that cell wall relaxation may be the main process of early berry enlargement induced by exogenous GA_3_. Cell wall relaxation genes (*EXPAs*, *XETs*) play a key role in GA_3_-induced grape fruit enlargement [16]. Our research also showed that after treatment with exogenous GA_3_, the expression of *VvEXPA8* and *VvEXPA11* was significantly upregulated (Figure 4). This shows that exogenous GA_3_ treatment upregulates the expression of genes related to cell wall relaxation, which in turn affects the expansion of cells and promotes the enlargement of early fruit development.

Expansins are a class of cell wall proteins that are involved in controlling cell enlargement and other developmental processes by regulating cell wall relaxation [49]. During tomato fruit enlargement, SlCRCa negatively regulates tomato fruit size by repressing the expression of *EXP* genes (such as *EXPA7* and *EXPA20*) [50]. In a recent study, it was found that the most frequently enriched cis-acting element (CRE) of the *VvEXPAs* promoter is G-box, which usually binds to transcription factors containing bZIP or bHLH DNA binding domains [51]. We cloned the promoters of *VvEXPA8* and *VvEXPA11* and analyzed their cis-acting elements and found that the promoters of *VvEXPA8* and *VvEXPA11* also contained G-box (Supplementary Table S3). Studies have shown that grape VvCEB1 is a specific bHLH transcription factor involved in the expansion of grape berry cells, and the upregulated expression of the *VvEXPAs* gene was detected in transgenic grape embryos overexpressing VvCEB1 [43]. In this study, we used EMSA and luciferase assay to prove that VvCEB1 can bind to the G-box element on the *VvEXPA8* and *VvEXPA11* promoters and activate its expression. At the same time, we also found that GA_3_ treatment promoted the up-regulation of VvCEB1 expression (Figure 6d), which indicates that VvCEB1 may be involved in the process of GA_3_ treatment to promote fruit cell expansion. As the response to the gibberellin response is mainly regulated by the DELLA protein, we further found that VvDELLA2 and VvCEB1 can interact, and the interaction of VvDELLA2 and VvCEB1 inhibits the expression of target genes *VvEXPA8* and *VvEXPA11*, indicating that the interaction of VvDELLA2-VvCEB1 inhibits the transcriptional activation activity of VvCEB1. We also found that VvDELLA2 binds to the C-terminal region of VvCEB1 instead of its DNA binding domain (Figure 6b,c), which indicates that the VvDELLA2-VvCEB1 interaction may change the conformation of VvCEB1. This is different from the known mode of the DELLA protein, which is that the DELLA protein interacts with the DNA binding domain of the transcription factor to prevent the transcription factor from binding to the cis-acting element on the target gene promoter, resulting in the inhibition of the expression of the target gene [30,31].

Our data strongly suggest that GAs produced upon pollination in the ovaries would mediate the degradation of VvDELLA2 in early fruits to relieve its inhibitory effect upon VvCEB1, which activates VvCEB1 on the downstream cell expansion genes *VvEXPA8* and *VvEXPA11* to finally promote early enlargement of grapes’ fruit. In contrast, in unpollinated ovaries, VvDELLA2 degradation would not occur due to low endogenous GA levels and, hence, VvCEB1 would be blocked to regulate *VvEXPA* genes. Next, the role of the VvDELLA2-VvCEB1-VvEXPAs module in mediating endogenous GAs regulation of early grape berry enlargement will be further investigated and confirmed.

In conclusion, our study explains the molecular mechanism of exogenous GA_3_ regulating the early enlargement of grape berries. Further analysis showed that the regulatory role of VvCEB1 in the process of GA_3_ regulating fruit enlargement is related to VvDELLA2, and the interaction between VvDELLA2 and VvCEB1 affects the early grape fruit enlargement. This study provides new clues about the mechanism of exogenous GA_3_ regulating fruit expansion, and lays a foundation for further investigations of the interplay among regulatory proteins in the process of grape fruit enlargement.

## 4. Materials and Methods

### 4.1. Plant Material

In this study, the grape cultivar ‘Fenghou’ (*V. vinifera* × *V. labrusca*) was used as experimental material and planted in a nursery in Wenquan Town, Beijing, China. If not stated otherwise, all treatments were carried out continuously for 3 years, and the grapevines were 15 years old at the time of initial treatment.

### 4.2. Pharmacological Treatments

The phytohormone GA_3_ (Sigma-Aldrich, Co., St.Louis, MO, USA), which can regulate grape growth, was used in this study to induce fruit development at a concentration of 0.084 mM [7]. The inflorescences participating in the experiment were divided into two groups, namely the emasculated group and the non-emasculated group, with 10 inflorescences treated in each group. The inflorescences of the emasculation treatment group were sprayed by 0.084 mM GA_3_ solution (GA_3_ was dissolved in a solution containing 94.9% water, 5% ethanol, and 0.1% tween 80), and the inflorescences of the non-emasculated group as controls were sprayed with the solvent without GA_3_.

Inflorescences or young fruits from each treatment were collected 2, 6, 10, 14, 22, and 30 days after flowering (DAF). A portion of the inflorescence or young fruits samples were used to make paraffin sections for microscopic observation, and these samples were fixed in FAA (FAA fixation solution consisted of 63% ethanol, 27% water, 5% glacial acetic acid, and 5% formaldehyde). The other parts of the inflorescence or young fruits samples were used for gene expression analysis; these samples were immediately frozen in liquid nitrogen after been collected and stored in a −80 °C ultra-low temperature refrigerator for future use.

### 4.3. Organizational Structure Observation and Statistical Analysis

The ovaries or young fruits fixed by FAA were subjected to ethanol gradient dehydration, ethanol, and n-butanol gradient transparency (the concentration ratio of ethanol to n-butanol was 3:1, 1:1, and 1:3, respectively, and finally transitions to pure n-butanol), and gradient wax immersion, and were then embedded in paraffin. The wax blocks were cut into sections (8 μm) using a tissue slicer. The thin section was stained with safranin and fast green staining methods, and, after sealing with neutral gum, an Olympus CX31 microscope (Tokyo, Japan) was used to take photos and observe. The diameter, number of cell layers, and cell area of the inner, middle, and outer pericarp in ovaries or young fruit from 2, 6, 10, and 14 DAF were measured using ImageJ software. The EXCEL software was used for data statistics, and the SPSS 26.0 software was used for data analysis.

### 4.4. Analysis Gene Expression with Quantitative Real Time PCR (qRT-PCR)

Total RNA of ‘Fenghou’ grape ovaries or fruits was extracted by the CTAB method, which was modified. The Reverse Transcription System (Promega, Madison, WI, USA) was used to synthesize cDNA from total RNA according to the manufacturer’s instructions. qRT-PCR was performed using 2 × NovoStart^®^ SYBR qPCR SuperMix (Novoprotein, Beijing, China). The internal control gene *VvUBQ* was used for gene expression analysis. The transcriptional expression level of genes was calculated by the 2^−ΔΔCt^ method [52]. All data analyses included three biological replicates, and the error bar represented the standard error (±SE) of the three replicates. Significance analysis was performed by Student’s *t*-test (* *p* < 0.05; ** *p* < 0.01; *** *p* < 0.001; ***** *p* < 0.00001). The primers and their sequences are listed in Supplementary Table S1.

### 4.5. Detection of Protein Interactions

#### 4.5.1. Yeast Two-Hybrid Assay

The yeast two-hybrid (Y2H) assay method refers to the Matchmaker GAL4-based Two-Hybrid System 3 in the *Yeast Protocols Handbook* (Clontech, Mountain View, CA, USA). The coding regions of *VvDELLA2* and *VvCEB1* were amplified and cloned into pGADT7 or pGBKT7 vectors, respectively. For analyzing functional domains required for the interaction between VvDELLA2 and VvCEB1, VvCEB1(NO.1), VvCEB1(NO.2), VvCEB1(NO.3), and full-length VvCEB1 fragments were amplified and cloned into pGBKT7, and a full-length VvDELLA2 fragment was amplified and cloned into pGADT7. Yeast AH109 cells were co-transformed with specific bait and prey constructs. SD-2 (-Leu/-Trp) and SD-4 (-Leu/-Trp/-His/-Ade) mediums containing 4 mg mL^−1^ X-α-gal were used to validate the interactions between different transformed combinations. Primers used for generating constructs for yeast two-hybrid assays are listed in Supplementary Table S2.

#### 4.5.2. Bimolecular Fluorescence Complementation

The full-length or segmented cDNA sequences of VvCEB1 and VvDELLA2 were cloned into pSPYNE or pSPYCE vectors to construct fusions expressing YFP^N^ and YFP^C^ (Supplementary Table S2). The fusion expression vectors were transformed into Agrobacterium tumefaciens and the mixture of two different plasmids of equal volume was transformed instantaneously into *N. benthamiana* leaves, as described by Schutze et al. [53]. The injected *N. benthamiana* plants were cultured for 3 days at 28 °C for 8-hour light/16-hour darkness conditions, and the fluorescence of green fluorescent protein (GFP) was observed in the area of *N. benthamiana* leaves injected with Agrobacterium using a confocal microscope (Olympus Fluoview FV1000).

#### 4.5.3. Electrophoretic Mobility Shift Assay

Sample preparation: A histidine (His) fusion protein containing the CDS region of *VvCEB1* was constructed using a PET-30a (+) vector (Supplementary Table S2) and transformed into *E. coli* BL21 (DE3) cells. These cells were induced overnight at 16 °C after adding IPTG, and the cells was broken by an ultrasonic cell crusher at low temperature, after which the protein was collected and purified using a nickel column (Biotechnology, Shanghai, China). The purified VvCEB1 protein was washed with 500 mM imidazole.

Probe preparation: Oligonucleotide probes were synthesized and labeled with 5′-biotin (Sangon Biotech, Shanghai, China), which were made from complementary oligonucleotides annealed at 72 °C for 30 min. The Chemiluminescent Biotin-labeled Nucleic Acid Detection Kit (Beyotime Biotechnology, Beijing, China) was used to perform the electrophoretic mobility shift assays (EMSA). The primers and their sequences are listed in Supplementary Table S3.

#### 4.5.4. Luciferase Reporter Assay

The promoters of VvEXPA8 and VvEXPA11 were independently constructed into the expression vector pCAMBIA 2300 to construct the reporter gene plasmids *pVvEXPA8:: LUC* and *pVvEXPA11:: LUC*. The cDNAs of *VvCEB1* and *VvDELLA2* were inserted into the plant constitutive expression vector pCAMBIA 1305.1 independently to construct the transgenes of *35S::VvCEB1* and *35S::VvDELLA2*.The primers and their sequences have been listed in Supplementary Table S2. The constructed vectors were transformed into Agrobacterium EHA105 cells independently, referring to Weigel and Glazebrook’s method [54]. The transformed Agrobacterium cells were cultivated to an OD600 of 1.0. We collected the bacteria after centrifuging the bacterial solution for 5 min, and then we washed the bacteria twice with 1/2MS liquid culture medium. The transformed cells were resuspended in a mixture containing 1 mM MgCl2, 1 mM MES-KOH, and 50 μM acetosyringone until it reached an OD600 of 1.0. The Agrobacterium solution to be tested was mixed so that the OD600 of each solution was 0.5, and the bacteria solution to be tested included *pVvEXPA8::LUC* + empty vector (1:1) and *35S::VvCEB1*+ *pVvEXPA8::LUC* (1:1); *pVvEXPA11::LUC* + empty vector (1:1) and *35S::VvCEB1* + *pVvEXPA11::LUC* (1:1); *35S::VvCEB1* + *pVvEXPA8::LUC* (1:1) and *35S::VvDELLA2* + *35S::VvCEB1* + *pVvEXPA8::LUC* (1:1:1); *35S::VvCEB1* + *pVvEXPA11::LUC* (1:1) and *35S::VvDELLA2* + *35S::VvCEB1* + *pVvEXPA11::LUC* (1:1:1). The mixed bacterial solutions were injected with a 1 mL syringe (with the needle removed) into the back of the *N. benthamiana* leaf (a fully extended leaf that has a growth period of about 1 month), and the injected plants were cultured at 24 °C for 3 days before sampling and observation. The transformed *N. benthamiana* leaves were spread out with 100 mM of luciferin (Promega, Madison, WI, USA), and were observed and imaged with the NightShade LB 985 In Vivo Plant Imaging System (Berthold, Germany) after being placed in the dark for 5 min; the detection was completed within 30 min. At least three independent biological replicates were performed for each experiment.

## 5. Conclusions

Our study provides new clues for the mechanism of GA_3_ regulating fruit enlargement. The data in this article elucidate the molecular mechanism of exogenous GA_3_ regulating early grape berry enlargement, and the interaction between VvDELLA2 and VvCEB1 is an important factor in promoting early grape fruit enlargement.

## Data Availability

All the data supporting the results of this research have been presented in this article and the Supplementary Materials.

## Supplementary Materials

The following supporting information can be downloaded at: https://www.mdpi.com/article/10.3390/ijms241914870/s1.
