# Supplementary figures and images for: Interaction of VvDELLA2 and VvCEB1 Mediates Expression of Expansion-Related Gene during GA-Induced Enlargement of Grape Fruit
Source: Int J Mol Sci. 2023 Oct 3;24(19):14870. doi: 10.3390/ijms241914870 (PMC10573625; doi:10.3390/ijms241914870)

## Slide 1
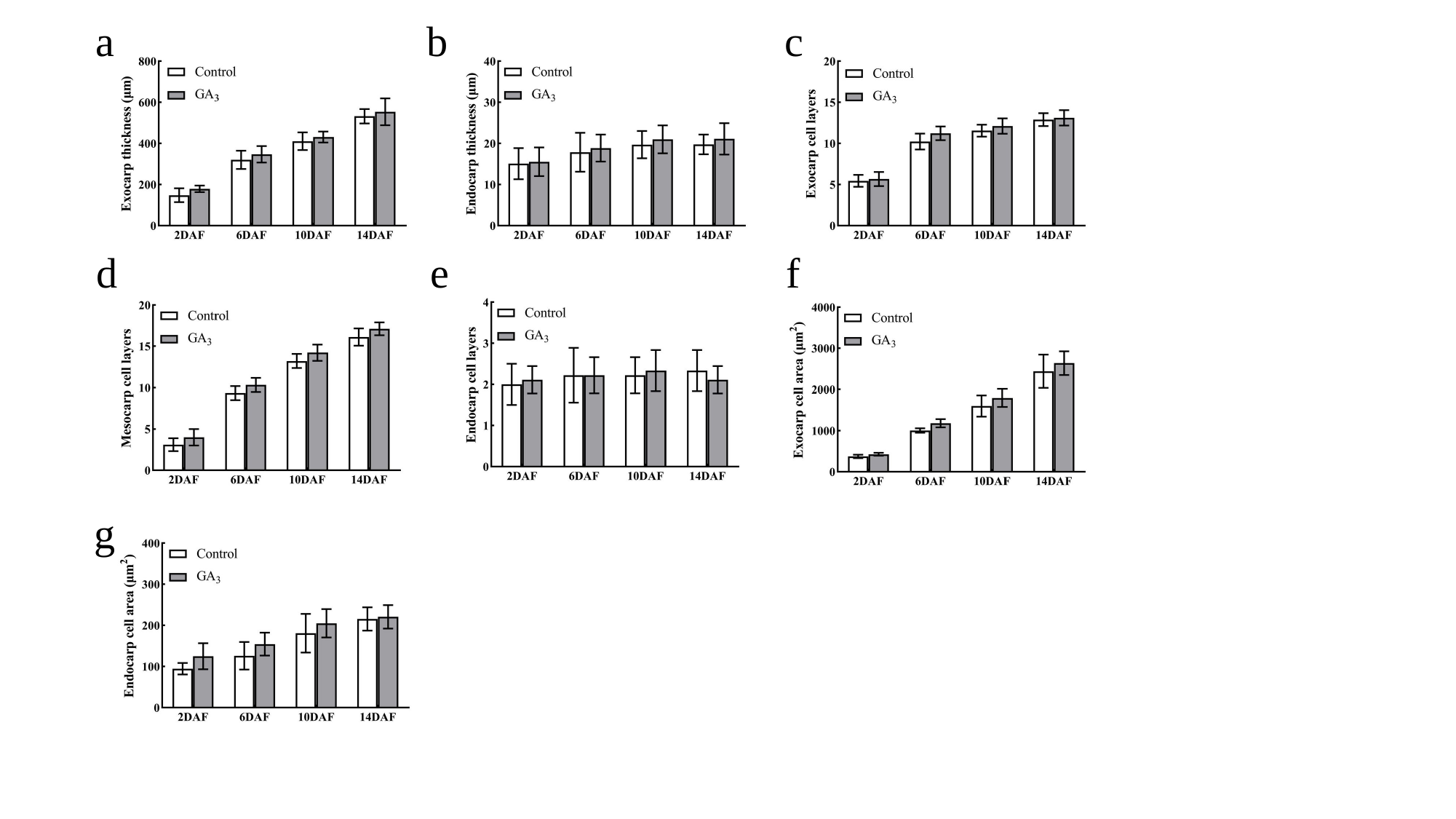

a
b
c
d
e
f
g

## Slide 2
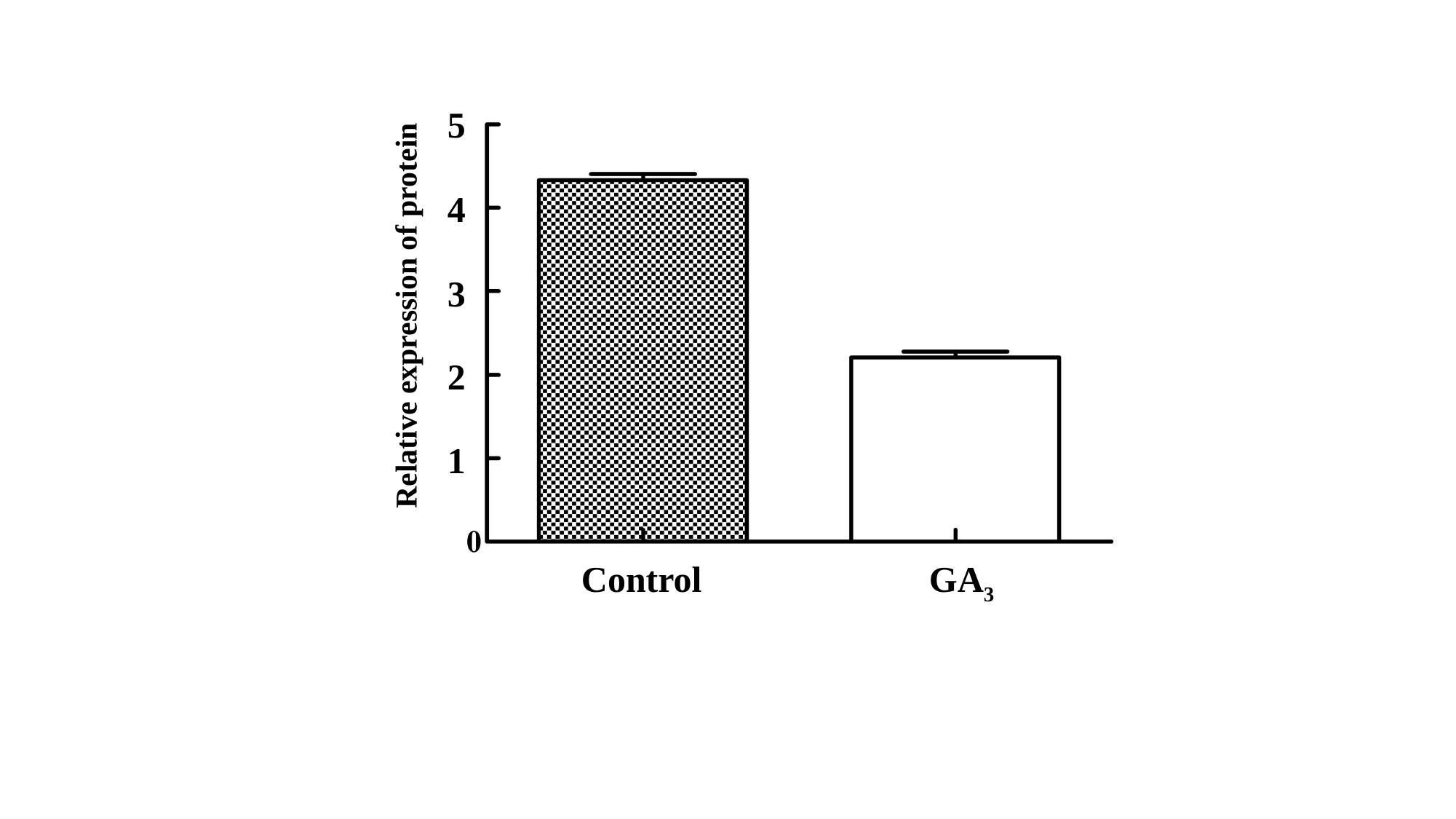

5
4
3
Relative expression of protein
2
1
GA3
Control

Supplement: Supplementary file 1 [file ijms-24-14870-s001.zip › Supplementary Figures.pptx]
